# Current Physical Therapy Practice in the Intensive Care Unit in Saudi Arabia: A Multicentre Cross-Sectional Survey

**DOI:** 10.1155/2020/6610027

**Published:** 2020-12-29

**Authors:** Mazen Alqahtani, Faizan Kashoo, Msaad Alzhrani, Fuzail Ahmad, Mohammed K. Seyam, Mehrunnisha Ahmad, Adel A. Alhusaini, Ganeswara Rao Melam, Syamala Buragadda

**Affiliations:** ^1^Department of Physical Therapy and Health Rehabilitation, College of Applied Medical Sciences, Majmaah University, Al Majmaah 11952, Saudi Arabia; ^2^Department of Nursing, College of Applied Medical Sciences, Majmaah University, Al Majmaah 11952, Saudi Arabia; ^3^Department of Rehabilitation Sciences, College of Applied Medical Sciences, King Saud University, Riyadh, Saudi Arabia

## Abstract

**Background:**

Early mobilisation of patients in the intensive care unit (ICU) is associated with positive health benefits. Research literature lacks insight into the current status of ICU physical therapy (PT) practice in the Kingdom of Saudi Arabia.

**Aim:**

To determine the current standard of ICU PT practice, attitude, and barriers.

**Methods:**

A questionnaire was e-mailed to physiotherapists (PTs) working in the hospital. The questions pertained to experience, qualification, barriers, and most frequently encountered case scenarios in the ICU.

**Results:**

The response rate was 28.1% (124/442). Frequent cases referred to the PTs were traumatic paraplegia (*n* = 111, 89%) and stroke (*n* = 102, 82.3%) as compared to congestive heart failure (*n* = 20, 16.1%) and pulmonary infections (*n* = 7, 5.6%). The preferred treatment of choice among PTs was chest physiotherapy (*n* = 102, 82.2%) and positioning (*n* = 73, 58.8%), whereas functional electrical stimulation (*n* = 12, 9.6%) was least preferred irrespective of the condition. Perceived barriers in the ICU PT management were of low confidence in managing cases (*n* = 89, 71.7%) followed by inadequate training (*n* = 53, 42.7%), and the least quoted barrier was a communication gap between the critical care team members (*n* = 8, 6.4%).

**Conclusion:**

PTs reported significant variation in the choice of treatment for different clinical cases inside ICU. The main barriers in the ICU setting were low confidence and inadequate training.

## 1. Introduction

Patients admitted to the intensive care unit (ICU) experience multiple complications, mandating multidisciplinary teamwork [1]. The physical therapist (PT) plays an important role in promoting short-term functional independence, reducing the hospital stay, improving quality of life, and early weaning of the patient from a ventilator [2]. The most common physical therapy (PT) treatment strategies in ICU include bed-side mobility exercises, ambulation, chest therapy, and weaning a patient from the ventilator [3].

The Diploma PT program was launched in the early 1980s in the Kingdom of Saudi Arabia (KSA). Subsequently, KSA has witnessed a marked rise in the number of universities offering Bachelor, Master's, and few Ph.D. in PT program [4]. The National Commission for Academic Accreditation and Assessment (NCAAA) [5] monitors and accredits programs offered at colleges and universities in KSA. Hospital sectors are divided into government regulated and privately regulated. Government hospitals are categorized into primary, secondary, and tertiary hospitals. The primary hospital provides basic medical care, whereas a tertiary hospital is equipped with latest and sophisticated medical equipment. The number of qualified PTs working in the hospitals and rehabilitation centres has grown considerably in the last few decades, reaching from 231 in 1994 to 2552 in 2018 as per the data published in World Physiotherapy webpage (https://world.physio/membership/saudi-arabia), and only 17.1% (440) are members of the Saudi Physical Therapy Association. This increased number of PTs in KSA is partially because of the mushrooming of universities and colleges across KSA.

A study conducted in South Africa reported that the PTs working in ICU lack standardized guidelines to practice in ICU [6]. However, the PTs in South Africa were involved in evidence-based practice [7]. There is no scientific literature available about the current PT practice in ICU in KSA hospitals. Therefore, the purpose of this study was to determine the current PT ICU practice and its associated barriers in KSA hospitals.

## 2. Methods

### 2.1. Study Design

The study was conducted through an online survey e-mailed to the PTs working in hospitals across KSA. The study started in August 2018 and ended in September 2019. Approval for the study was obtained from the Institutional Review Board of Majmaah University. The survey along with the consent form was sent to each of the PTs directly or to the head of departments of hospitals for dissemination.

### 2.2. Subjects

All PTs working in KSA were eligible to participate in the survey. Participation in this survey was voluntary, and participants did not receive any incentives for participation. E-mail of the participants was obtained from the hospital webpage or by contacting the head of the physical therapy department of the hospital. Informed consent forms were sent to the participants through an e-mail containing all the information related to the survey and contact details of the corresponding author. Nonresponsive participants were reminded every two weeks for two months. In case, the respondent did not respond after four consecutive reminders, further communication was stopped.

### 2.3. Survey Development

This current study adopted a questionnaire developed by Hodgin et al. [8]. Cronbach's alpha for the reliability of the questionnaire was 0.843. Prior permission to use the questionnaire was obtained from the developers of the questionnaire. The survey questions were inserted into the Google survey tool (https://surveys.google.com/forms) unaltered (English language).

The survey comprised of three sections: the first section contained 7 general items related to demographic data and description of PT practice in ICU, the second session comprised of 6 scenario-based questions, and the third session consisted of open-ended questions related to barriers to practice, training, and evidence-based practice. The survey consisted of a Likert scale ranging from 1 to 10 (where 1 was “very unlikely” and 10 was “very likely”). The most common PT treatment included cardiopulmonary physical therapy/“Chest Physiotherapy” (includes postural drainage, chest mobilization and manipulation, and weaning a patient from the ventilator), passive range of motion exercises (passive joint mobilisation and ankle-toe movements to avoid deep vein thrombosis), positioning (includes position changes to improve pulmonary capacity and to avoid pressure sore), exercises (includes aerobic or resisted exercises started by the patient with the help of the PT), functional activities (includes bed mobility, balance training, transfer training, and ambulation), and functional electrical stimulation (FES) (includes placing the electrodes on the particular area intended to assist paralysed muscle to complete a functional task). The final question after every case scenario was to select one among the most effective PT treatments.

### 2.4. Survey Analysis

The data obtained from the Google online survey were saved on Microsoft Excel™ 2018. The data were analyzed by SPSS software (version 19.0, IBM Corp, New York, USA). Demographic data were analyzed using descriptive statistics as mean and standard deviation or percentages. Open-ended answers were sorted out into five common categories, and the remaining questions were analyzed using SPSS. Fischer exact and Kruskal–Wallis (one-way analysis of variance) were used to analyze the data. The level of significance was set at 0.05 with the 95% confidence interval.

## 3. Results

### 3.1. General Demographic

Four hundred and forty-two (442) PTs were contacted through e-mail. The authority concerned in the hospital provided the number of PTs working in the department and their contact details. One hundred and twenty-four (124) PTs completed the questionnaire with an overall response rate of 28.1%. Out of 124 PTs, 83 (66.9%) were working in tertiary hospitals (TH), 15 (12.1%) in secondary hospitals, 14 (11.2%) in private hospitals, and 12 (9.6%) in primary hospitals. Forty-eight (38.7%) of PTs in TH had over 10 years of PT experience as compared to PTs working in the primary hospitals and secondary government regulated hospitals. Thirty-nine (31.5%) PTs working in TH had 10 years of ICU experience as compared to primary (*n* = 18, 14.5%) and secondary (*n* = 32, 25.8%) hospitals. The mean average working hours for all the health sectors was 8 hours/day ranging from 8.7 hrs/day in private hospitals to 6.8 hrs/day in secondary hospitals (Table 1).

### 3.2. Referral and Preferred PT Approach in ICU

The probability of patients referred to PTs varied with the clinical case (highest 89.5% and 82.3% for traumatic paraplegia and stroke, respectively, and lowest 5.6% for pulmonary infections) (*P* < 0.001) (Table 2).

Chest physiotherapy was preferred in all the clinical cases with the highest mean value of 9.79 ± 0.19 for respiratory failure and 8.71 ± 1.66 for patients intubated with pulmonary infections and least preferred in road traffic accidents (2.04 ± 1.57). Functional electrical stimulation was least preferred with the highest mean value ranging from 3.06 ± 1.66 for stroke to 1.44 ± 0.87 for road traffic accidents (Table 3).

One-way ANOVA was used to compare means of clinical case scenario for each of the PT treatment. Results showed that there was a significant difference in the preference of the treatment choice such as cardiopulmonary physical therapy, range of motion exercises, positioning, and functional activities among the different clinical scenarios. Conversely, exercises (*P*=0.35) and functional electrical stimulation (*P*=0.111) were provided to all patients irrespective of clinical scenarios (Table 4).

### 3.3. Barriers of Practice

Based on the response from the participants, barriers to PT practice are categorized into four types.

1. Staffing: 66.1% (*N* = 82) of the participants responded that the staff required is not sufficient
2. Training: 57.2% (*N* = 71) of the participants felt that additional training in ICU management would improve their quality of care
3. PT practice outcome in ICU: the patient outcome after the PT session is one of the biggest motivating factors among PTs working in ICU. 41.9% (*N* = 52) of PTs reported appreciation and encouragement by the associated critical care team as essential motivating factors among PTs.
4. PT's work as a team: 20.1% (*N* = 25) of participants felt the communication gap between the team members in ICU management of the patient. A regular case discussion among various health professionals involved in ICU management of the patient is essential to improve the quality of patient care.

## 4. Discussion

The aim of this study was to report the current status of PT practice in ICU in KSA. This current study is able to put forward the current scenario of practice in ICU in terms of the use of the PT modalities/therapeutics for a specific case, demographic data, education, and barriers to practice in KSA.

Systematic reviews [9, 10] reported that the common treatment strategies used by therapists in the hospital were positioning, manual hyperinflation, mobilisation, percussions, and vibrations, suctioning, exercises, and continuous rotational therapy. The amount of use of these treatment strategies depended on the clinical case [11]. Recent reviews also reported that there is a need to provide robust evidence of the efficacy of PT interventions [12] in ICU, and the current evidence is limited to levels C and D [13].

In this current study, the most preferred treatment among PTs was chest physiotherapy (54%) irrespective of the clinical cases. The efficacy of chest PT is well defined and includes combination of postural drainage, manual hyperinflation, percussion, and vibration [14]. Similar ICU practice is followed in the USA [15], Europe [16], and Asia [17]. However, a study conducted in Greece reported variation in PT procedures among PTs regarding early mobility and respiratory PT [18]. A study in Nepal reported that PTs practice was restricted to only chest physiotherapy [17].

Positioning was the second most common treatment preferred by PTs in ICU in KSA. A recent study [19] reported that a high percentage of therapists agree that positioning is helpful to prevent bedsores and improve patients' comfort. A cross-sectional study [20] among nurses and physicians reported that positioning is an important intervention to reduce the chances of bedsores among patients admitted in ICU. A study by Norrenberg and Vincent had reported a similar PT practice across European countries [16].

Functional activities (also includes bed mobility and gait training) and therapeutic exercises were moderately preferred by 46% and 39% of PTs, respectively, although strong efficacy of these PT techniques is reported and is the preferred procedure in the USA [21] and many European countries [22]. A systematic review conducted on such clinical practice guidelines support early mobilisation in ICU [23].

Functional electrical stimulation was the least preferred (13%) treatment in ICU in the KSA. Although a study reported an increase in muscle strength with FES in critically ill patients [24], the possible reason for the least use of FES was the lack of knowledge among PTs about its efficacy and training [25].

The barriers to PT practice found in this survey were categorized into four categories. Participants in this study reported that inadequate staff (66%), inadequate ICU training (57%), lack of motivation (41.9%), and lack of communication between the critical care team (21.1%) were the main barriers. A study conducted among Chilean physiotherapists working in ICU reported low specialist training among PTs in ICU settings [26]. A similar study conducted in the United States [27] reported that the prioritization of policies, less number of qualified staff, inadequate training, and inadequate consultation were the main barriers to PT practice in ICU. In this study, 57% of PTs reported a lack of training in the ICU physical therapy management during their education. Most of the PTs responded that they learnt ICU PT management from their seniors and coworkers. Research conducted in Jordan reported that the main barriers were inadequate training, insufficient staffing, inadequate perceived importance, and consultation with the PTs working in ICU [28]. A similar trend was reported in Sri Lanka regarding inadequate staffing and training in ICU among PTs [29].

Most participants showed a neutral response towards the prioritization of service, PT consultation, and the importance of PT sessions in ICU as perceived by other team members. This is despite strong evidence that PT sessions in ICU improve quality of life, shorten the stay in ICU, and reduce financial cost. A study reported that 24 hours availability of PT service as compared to 12 hours would significantly reduce ICU cost [30].

In order to overcome the barriers and improve the quality of care in ICU PT practice, it is recommended that the academic program must include course learning outcomes related to knowledge and psychomotor skills involved in the ICU PT management [31–33]. A study conducted in Australia and New Zealand developed a 132-item consensus framework for a minimum standard of PT practice in ICU [34]. Similarly, a list of clinical standards of practice in ICU has been developed by South African [35], UK [36], and Japanese PTs [37].

The study has the following limitations:

1. The response rate from the participants was low (28.1%)
2. A study conducted to validate the knowledge, behavior, and skill of the PTs working in ICU to be used in designing the residency and fellowship program [38]. This present study although was limited in exploring certain characteristics of the ICU, but can be useful to better design the Bachelor and Master's curriculum to suit the present need.
3. Length of the survey, the six scenarios, and question associated with each scenario makes the questionnaire lengthy and time-consuming
4. The self-reported questionnaire is always at the risk of response bias and easily gets influenced by the circumstances around the participants

Future studies must explore the correlation between the years of ICU experience and quality of care. Studies may also explore the adherence to clinical guidelines in ICU.

## 5. Conclusion

The study revealed the following facts about ICU PT management.

1. PTs had low confidence in managing patients in ICU because of inadequate training
2. Lack of academic training, low confidence, and difficulty in interpreting values on the ICU monitor were the main barriers to practice
3. Chest physiotherapy and positioning were the preferred modes of treatment by a physiotherapist working in ICU
4. Functional mobility training and aerobic exercises were moderately utilized in ICU

## Figures and Tables

**Table 1:** Demographic data of the participants.

	All	Government regulated			Privately regulated
	*n* = 124	Primary hospitals	Secondary hospitals	Tertiary hospitals	Private hospital
Age years (SD)	28.5 (6.9)	34.4 (3.7)	29.4 (5.3)	32.7 (6.1)	25.9 (6.9)

Experience as PT, *n* (%)					
1–3	19 (15.3%)	4 (3.2%)	0	13 (10.5%)	2 (1.6%)
4–6	13 (10.4%)	1 (0.8%)	3 (2.4%)	4 (3.2%)	5 (4.0%)
7-8	19 (15.3%)	4 (3.2%)	2 (1.6%)	12 (9.7%)	1 (0.8%)
9-10	16 (12.9%)	2 (1.6%)	5 (4%)	6 (4.8%)	3 (2.4%)
>10	57 (45.9%)	1 (0.8%)	5 (4%)	48 (38.7%)	3 (2.4%)
Total	124 (100%)	12 (9.6%)	15 (12.1%)	83 (66.9%)	14 (11.2%)

ICU experience as PT, *n* (%)					
No experience	11 (8.8%)	1 (0.8%)	6 (4.8%)	1 (0.8%)	3 (2.4%)
1–3	12 (9.6%)	2 (1.6%)	7 (5.6%)	2 (1.6%)	1 (0.8%)
4–6	4 (3.2%)	0	1 (0.8%)	2 (1.6%)	1 (0.8%)
7-8	19 (15.3%)	3 (2.4%)	3 (2.4%)	11 (8.9%)	2 (1.6%)
9-10	11 (8.8%)	1 (0.8%)	2 (1.6%)	5 (4%)	3 (2.4%)
>10	67 (54%)	11 (8.9%)	13 (10.5%)	39 (31.5%)	4 (3.2%)
Total	124 (100%)	18 (14.5%)	32 (25.8%)	60 (48.3%)	14 (11.2%)

Working hours (mean)	8	8.4	6.8	8.1	8.7

*Note*. PT, physiotherapist; *n,* number; SD, standard deviation; %, percentage; ICU, intensive care unit.

**Table 2:** Frequency of clinical cases referred to physical therapy.

	Referred to PT, *n* (%)			
Clinical cases	<25%	25–50%	51–75%	75–90%
Major stroke	0	10 (8.1%)	12 (9.7%)	102 (82.3)
RF	8 (6.4%)	30 (24%)	41 (32.8%)	45 (36%)
Traumatic paraplegia	3 (2.4%)	1 (0.8%)	9 (7.3%)	111 (89.5%)
CHF	4 (3.2%)	13 (10.4%)	68 (54.8%)	39 (31.4%)
PI	26 (61.3)	76 (61.3)	15 (12.1)	7 (5.6%)
RTA	6 (4.8%)	67 (54.1%)	31 (25%)	20 (16.1%)
Total	47 (6.3%)	197 (26.4%)	176 (23.6%)	324 (43.5%)

RF, respiratory failure; CHF, congestive heart failure; COPD, chronic obstructive pulmonary disease; PI, pulmonary infection; RTA, road traffic accident.

**Table 3:** Preferred physical therapy approach in ICU.

Common clinical cases in ICU	Cardiopulmonary physical therapy	Passive range of motion exercises	Positioning	Exercises	Functional activities	Functional electrical stimulation
Major stroke	6.00 ± 1.10	9.31 ± 1.69	7.60 ± 1.39	9.58 ± 1.67	5.96 ± 1.86	3.06 ± 1.65
Respiratory failure	9.79 ± 0.19	6.15 ± 1.14	6.35 ± 0.95	3.46 ± 1.84	3.81 ± 2.03	2.33 ± 1.26
Vertebral fracture	5.41 ± 1.91	3.73 ± 1.90	4.60 ± 1.81	3.35 ± 1.75	3.17 ± 1.92	1.50 ± 0.96
Congestive heart failure	6.02 ± 1.43	3.38 ± 1.76	4.52 ± 1.74	3.87 ± 1.72	3.73 ± 1.86	1.50 ± 0.87
Intubated with pulmonary infection	8.71 ± 1.66	2.98 ± 1.81	4.40 ± 1.79	4.02 ± 1.84	2.90 ± 1.49	1.46 ± 0.90
Road traffic accident	2.04 ± 1.57	3.10 ± 1.74	3.69 ± 2.09	3.67 ± 1.77	3.65 ± 1.79	1.44 ± 0.87

The values are mean ± SD of a Likert score (from 1 (very unlikely) to 10 (very likely).

**Table 4:** Effect of clinical cases scenario on preference of physical therapy treatment.

	*n*	Mean ± SD	*P*
Cardiopulmonary physical therapy			
Major stroke	161	6.00 ± 1.10	0.002
Respiratory failure	173	9.79 ± 0.19	
Vertebral fracture	123	5.41 ± 1.91	
Congestive heart failure	79	6.02 ± 1.43	
Intubated with pulmonary infection	98	8.71 ± 1.66	
Road traffic accident	56	2.04 ± 1.57	

Range of motion exercises			
Major stroke	161	9.31 ± 1.69	0.001
Respiratory failure	173	6.15 ± 1.14	
Vertebral fracture	123	3.73 ± 1.90	
Congestive heart failure	79	3.38 ± 1.76	
Intubated with pulmonary infection	98	2.98 ± 1.81	
Road traffic accident	56	3.10 ± 1.74	

Positioning			
Major stroke	161	7.60 ± 1.39	0.04
Respiratory failure	173	6.35 ± 0.95	
Vertebral fracture	123	7.60 ± 1.81	
Congestive heart failure	79	4.52 ± 1.74	
Intubated with pulmonary infection	98	7.40 ± 1.79	
Road traffic accident	56	8.69 ± 2.09	

Exercises			
Major stroke	161	5.58 ± 1.67	0.35
Respiratory failure	173	3.46 ± 1.84	
Vertebral fracture	123	3.35 ± 1.75	
Congestive heart failure	79	3.87 ± 1.72	
Intubated with pulmonary infection	98	4.02 ± 1.84	
Road traffic accident	56	3.67 ± 1.77	

Functional activities			
Major stroke	161	5.96 ± 1.86	0.02
Respiratory failure	173	3.81 ± 2.03	
Vertebral fracture	123	3.17 ± 1.92	
Congestive heart failure	79	3.73 ± 1.86	
Intubated with pulmonary infection	98	2.90 ± 1.49	
Road traffic accident	56	3.65 ± 1.79	

Functional electrical stimulation			
Major stroke	161	3.06 ± 1.65	0.111
Respiratory failure	173	2.33 ± 1.26	
Vertebral fracture	123	1.50 ± 0.96	
Congestive heart failure	79	1.50 ± 0.87	
Intubated with pulmonary infection	98	1.46 ± 0.90	
Road traffic accident	56	1.44 ± 0.87	

ANOVA test (*P* < 0.05) is used to analyze the difference in choice of treatment in different clinical cases. SD, standard deviation; *n,* number of responses.

## Data Availability

The data used to support the findings of this study are available by the corresponding author upon request. The preprint of the article is available in the following link: https://www.researchsquare.com/article/rs-8291/v1.
